# Exploring the potential of black tea based flavonoids against hyperlipidemia related disorders

**DOI:** 10.1186/s12944-018-0688-6

**Published:** 2018-03-27

**Authors:** Ali Imran, Masood Sadiq Butt, Muhammad Sajid Arshad, Muhammad Umair Arshad, Farhan Saeed, Muhammad Sohaib, Rizwan Munir

**Affiliations:** 1 Institute of Home and Food Sciences, Government College University, Faisalabad, 38040 Pakistan; 2 National institute of Food Science and Technology, University of Agriculture Faisalabad, Faisalabad, 38040 Pakistan; 3 Department of Food Science and Human Nutrition, University of Veterinary and animal Sciences, Lahore, 54000 Pakistan; 4 Department of Statistics, Government College University, Faisalabad, 38040 Pakistan

**Keywords:** Hyperlipidemia, Dietary interventions, Flavonoids, Thearubigins

## Abstract

**Background:**

In recent decade, Hyperlipidemia related disorders like obesity, hypercholesterolemia and diabetes are considered as the leading killers for mankind. Fundamental nexus between nutrition and health diverting the consumers focus towards plant based natural products as a remedy against various metabolic syndrome. Considering this, present study was conducted to explicate the role of black tea polyphenols such as Theaflavins and thearubigins therapeutic potential to tackle targeted maladies especially oxidative stress related disorders like hypercholesterolemia and diabetes.

**Methods:**

The mandate of current investigation was to explore the hypoglycemic and hypocholestrolemic perspective of isolated theaflavin and thearubigins through a model feeding trial. For the purpose, theaflavin & thearubigins were isolated from black tea through solvent partition method and utilize to form three types of nutraceutical drinks (theaflavin, thearubigins & theaflavin + thearubigins based) alongside control to be further utilized in bioefficacy trial. In bioefficacy trial, three types of independent studies were design on the bases of diet by involving 20 male wistar rats in each study (5 for each group). In study I, normal diet was administrated while, in study II & III high cholesterol and high sucrose diet was given, respectively along with prepared nutraceutical drinks to synchronize their therapeutic effect for a period of 56 days. At the termination of trial, Feed & drink intakes, body weight, total cholesterol, LDL, HDL, triglycerides, glucose and insulin levels were measured.

**Results:**

The results indicated reduction in cholesterol, LDL and triglycerides levels of experimental rats in all studies with significant increase in HDL. In this context, theaflavin based drink imparted maximum reduction in cholesterol (3.75, 11.03 & 10.39%), LDL (3.84, 14.25& 10.84%) & triglycerides (2.99, 8.54 & 6.65%) in respective studies compared to thearubigins and theaflavin + thearubigins based drinks. However, theaflavin+ thearubigins based drink caused highest glucose decline and maximum insulin increase in all studies as compared to other nutraceutical drinks. The reported value for the insulin increase were 13.02 ± 1.02 & 14.55 ± 1.13, 10.09 ± 0.15 & 11.59 ± 0.86 for Hyperglycemic and Hypocholestrolemic rats respectively compared to control (7.84 ± 0.45 & 9.10 ± 0.41) for study I and II.

**Conclusions:**

In the nutshell, theaflavin and thearubigins based dietary interventions are helpful to alleviate the hypercholestrolemia and hyperglycemia and should be promoted as parallel therapy to combat these disorders.

## Background

Changing life style, poor diet pattern and lack of physical activity have amplified the onset of various metabolic malfunctioning like hyperlipidemia and hyperglycemia and cardiovascular complications. Diabetes is a metabolic syndrome considered as a third “killer” of mankind along with cancer and coronary complications. Currently, Pakistan is holding 6th position in terms of diabetic patients that are estimated to be about 13.85 million in the year 2030 [1]. Management of blood cholesterol continues to be a cardinal issue in cardiovascular diseases (CVD). Hypercholesterolemia and LDL oxidation played a key role in the onset of atherosclerosis and related complications. Fundamental nexus between nutrition and health diverting the consumers focus towards plant based natural products as a remedy against various metabolic syndromes. In diet based regimen, functional/nutraceutical foods are gaining core attention of the nutritionists to be a therapeutic device against the maladies [2]. No doubt, medicines are inevitable for curing various physiological disorders nevertheless, high treatment cost predominantly in the developing countries along with associated side effects demanding some other rationale approaches to meet the perils. In this scenario, black tea being a functional beverage is important due to its accessibility, low cost and allied therapeutic claims [3]. Extensive studies have suggested that black tea consumption provides numerous health benefits mainly attributed to its polyphenols especially theaflavin and thearubigins [4]. These bioactive moieties have potential against hypercholesterolemia and hyperglycemia along with oncogenic and renal modulating perspectives owing to their high antioxidant capacity.

Globally, tea is a popular beverage after water made from tea plant leaves. The historians have linked its consumption almost 5000 years back [5]. Tea is mainly divided into three distinct types i.e. black, green and oolong differed in terms of processing method and chemical profile. Green tea accounts for approximately 20% of total tea production, consumed primarily in East and South East Asia. Contrarily, black tea that occupies approximately 78% of the world share is consumed mainly in North America, Europe and North Africa. Theaflavin is a group of four compounds constitute about 3–6% of the black tea polyphenols. Structurally, it consists of hydroxy-substituted benzotropolone ring synthesized by condensation of catechins in their hydroxylated B rings attached with benzotropolone catechins [6]. Being a natural antioxidant, theaflavin exhibits radical scavenging and chelating properties owing to the presence of hydroxyl group along with gallic acid moiety. Furthermore, it prevents lipid preoxidation as well as induces activation in different antioxidant enzymes like glutathione-S-transferases and catalase. Besides, theaflavin provides particular characteristics to the tea like brisk, taste and color [7]. Likewise, thearubigins are the copious group of black tea polyphenols often termed as polymeric black tea substances (PBS). They also impart significant effect on tea taste due to their hydrophilic nature and activation of phase II enzymes [8]. Likewise, thearubigins has ability to prevent DNA from mutation [9]. Theaflavin & thearubigins have singlet oxygen quenching ability they act as safeguard against oxidative stress thereby effective in the maintenance of cardiac health and cancer care. Theaflavis and other tea polyphenols have showed tendency to inhibit the expressions of key obesity related targets like pancreatic lipase (PL) that plays a central role in fat metabolism. Moreover, it stimulates lipolysis associated with the induction of mitochondrial uncoupling proteins and AMPK–FoxO3A–MnSOD pathway in 3 T3-L1 adipocytes that are helpful in obesity management [10, 11]. Likewise, tea polyphenols especially theaflavin impart inhibitory effect on starch-hydrolysing enzymes like α-amylase and α-glucosidase thus helpful for the management of postprandial hyperglycemia in diabetic complications [12]. Considering the therapeutic consensus of black tea polyphenols, the mandate of current study was to probe the hypocholestrolemic & hypoglycemic potential of theaflavin & thearubigins based functional drinks through a model feeding trial.

## Methods

### Isolation of the Theaflavin and thearubigins

Theaflavin and thearubigins were isolated locally cultivated black tea variety Qi-Men procured from the National Tea Research Institute (NTRI), Shinkiari, Mansehra. The reagents (analytical and HPLC grade) and standards were purchased from Merck (Merck KGaA, Darmstadt, Germany) and Sigma-Aldrich (Sigma-Aldrich Tokyo, Japan). For efficacy trial, Male Sprague Dawley rats with initial body weight of 128 ± 10.25 g/rat were housed in the Animal Room of NIFSAT. For biological assay, diagnostic kits were purchased from Sigma-Aldrich, Bioassay (Bioassays Chemical Co. Germany) and Cayman Chemicals (Cayman Europe, Estonia).

### Theaflavin and thearubigins extraction yield estimation

Estimation of theaflavin (TF) and thearubigins (TR) of the resultant extracts was carried out following the protocol of [13]. Briefly, in a separating funnel equal amount of extract and iso-butyl methyl ketone (IBMK) were added. After separation, resultant organic layer was diluted with 9 mL of ethanol, absorbance (380 nm) was calculated and considered as A. In the next step, 10 mL of organic phase was diluted by adding 10 mL of Na_2_HPO_4_ (2.5%). The separated layer was again diluted with ethanol, measured absorbance at same wavelength and termed as B. Lastly, butanol treated aqueous phase was eluted with 9 mL of ethanol and measured absorbance at 380 nm, named as C.

$$ \mathrm{TF}\ \left(\%\right)=4.313\times \mathrm{C} $$

$$ \mathrm{TR}\ \left(\%\right)=13.643\times \left(\mathrm{A}+\mathrm{C}-\mathrm{B}\right) $$

### HPLC quantification of theaflavins

Isolated theaflavin were quantified through HPLC (PerkinElmer, Series 200, USA) using C_18_ column (250 mm × 4.6 mm, 5.0 μm particle size). A 10 μL aliquot of sample was taken through auto sampler (WISP Model 710) and maintained the column temperature 40 °C throughout the analysis. During theaflavin quantification, mobile phase was comprised of acetonitrile, ethylacetate and 0.05% phosphoric acid in ratio of 21:3:76. The flow rate was maintained at 1 mL/min followed by quantification with U*V*/*v*is detector (model 481, wavelength at 278 nm).

### Dietary interventions preparation

Four types of Nutraceutical experimental drinks were prepared, the first (T_1_) contained theaflavin (500 mg/500 mL) whilst other (T_2_) enriched with thearubigins (500 mg/500 mL). The third sample (T_3_) was prepared by combining both functional ingredients (250 + 250 mg/500 mL) to evaluate their synergistic effect along with control (T_0_) for comparison purpose. All functional drinks were prepared by adding aspartame, citric acid, sodium benzoate, carboxy methyl cellulose (CMC), food grade color and flavor. Active ingredients including theaflavin and thearubigins were added in the respective drinks T_1_ and T_2_ @ 500 mg/500 mL, whilst in T_3_ half of the dose (250 + 250 mg/500 mL) of each was used. Theaflavin and thearubigins were isolated through solvent partition method [10]. Initially, extracts except water based were concentrated through Rotary Evaporator (Eyela, Japan) and after filtration subjected to solvent partition using chloroform, ethyl acetate and butanol. Theaflavin and thearubigins rich fractions were separated followed by rotary evaporation and freeze drying (CHIRST, Alpha 1–4 LD plus, Germany).

### In vitro pancreatic lipase inhibitory activity

To evaluate the effectiveness of black tea flavonoids against lipid abnormalities, the in vitro pancreatic lipase inhibitory activity was monitored by adapting the guidelines of Kubdi et al. [14]. In brief, p-nitrophenyl palmitate (p-NPP) utilized as a substrate which under the influence of enzyme release substrate. The enzyme under the reaction conditions release p-nitrophenol after the hydrolyses p-NPP and activity is monitored at 410 nm. To create the reactions, serial dilutions of study compounds were made in DMSO (25–600) mg/mL. Furthermore, Lipase (0.1 mg) was added in Tris-buffer (50 mM, pH 8). Then mixture was stirred for 15 min and centrifuged at 2000 rpm for 10 min. The clear supernatant was recovered. Afterwards, 0.5 Ml of lipase solution was mixed with double amount of theaflavin and thearubigins of their respective solvents and incubated for 30 min at 37 c then added 0.5 mL of substrate p-NPP and note absorption at 410 nm and % inhibition was calculated by adapting the following equation

$$ \%\mathrm{Activity}=\frac{1}{2}\mathrm{Ac}-\mathrm{As}=\mathrm{Ac}\_\times 100 $$

where Ac and As are the absorbance of control and sample, respectively.

### Biological assay

To conduct the biological trial, the standard animal experiment guidelines were adapted. Initially ethical approval was obtained from the Animal care committee (ACC) of National Institute of Food Science and Technology (NIFSAT) University of Agriculture, Faisalabad. For the purpose, 130 male Sprague Dawley rats with initial body weight of 128 ± 10.25 g/rat were housed in the Animal Room of NIFSAT, University of Agriculture, Faisalabad. The rats were acclimatized by feeding on basal diet (AIN-76A) for a period of one week. The environmental conditions were control throughout the trial like temperature (23 ± 2 °C) and relative humidity (55 ± 5%) along with 12-h light-dark period. At the initiation of study, ten rats were sacrificed to establish the baseline trend. During efficacy trial, three types of studies were conducted independently by involving normal, hypercholesterolemic & hyperglycemic rats. In Study I, rats were fed on normal diet whereas in study II & III high cholesterol and high glucose diets were administrated, respectively. Each study comprised of four groups of rats 05 in each. Accordingly, four types of drinks i.e. control, theaflavin enriched, thearubigins supplemented and a combination of theaflavin and thearubigins were prepared considering the stability of the active ingredients and given to the representative groups. During the eight weeks trial, instantaneous administration of nutraceutical drinks to experimental rats was ensured to assess their therapeutic role. The drinks were administrated @ 250 ml for each group and control group received the drink that contained all other ingredients except for theaflavin and thearubigins for a period of 56 days (Table 1). At the termination of the study, overnight fasted rats were decapitated and blood was collected. For serum collection, blood samples were subjected to centrifugation using centrifuge machine @ 4000 rpm for 6 min. The respective sera samples were examined for various biochemical assays by using Microlab 300, Merck, Germany. Different biochemical parameters including total cholesterol, LDL, HDL, triglyceride, glucose, insulin levels and serum antioxidant enzymes. The details of these studies are herein;

**Table 1:** Diets and functional drinks plan

Studies	(Study I) Normal rats				(Study II) Hypercholesterolemic rats				(Study III) Hyperglycemic rats			
	Normal diet				High cholesterol diet				High sucrose diet			
Groups	1	2	3	4	1	2	3	4	1	2	3	4
Drinks	T_0_	T_1_	T_2_	T_3_	T_0_	T_1_	T_2_	T_3_	T_0_	T_1_	T_2_	T_3_

Each group consist of 5 male wistar rats in each (20 for one study). All studies were independent and in all animal were provided 250 mL of respective drink for a period of 56 days alongside respective diets

T_0_: Control

T_1_: Drink containing theaflavin

T_2_: Drink containing thearubigins

T_3_: Drink containing theaflavin+thearubigins

### Study I: Normal rats

In this study, rats were divided in to four homogeneous groups fed on normal diet along with provision of respective functional drink. The experimental diet was formulated using corn oil (10%), protein (10%), corn starch (66%), cellulose (10%), and mineral (3%) and vitamin mixture (1%). Following similar approach, three other studies were conducted to find out the impact of functional drinks against respective diets i.e. high cholesterol & high sucrose enriched to correlate with the lifestyle related disorders.

### Study II: Hypercholesterolemic rats

In study II, high cholesterol diet i.e. 1.5% of cholesterol along with cholic acid 0.5% was given to the normal rats to induce hypercholesterolemia. Periodic examination of rats was carried out to assess the induction of hypercholesterolemia. The functional drinks were provided to the rats concurrently to synchronize their effect on the respective group.

### Study III: Hyperglycemic rats

In study III, high sucrose diet containing 40% sucrose was given to induce hyperglycemia in rats and determined its effect on serum glucose and insulin levels. Besides, effect of functional drinks on the induced syndrome was measured in each group at the termination of the study.

### Physical parameters

Feed intake was measured daily by subtracting the spilled diet from the total diet during the whole trial (11). The functional drink intake of each group was also recorded daily by monitoring the differences in the graduated bottles.

### Body weight gain

Gain in body weight of experimental groups was measured weekly throughout the study period to monitor any suppressing effect of experimental drinks on this trait.

### Serum lipid profile

Serum cholesterol level was determined using CHOD–PAP method [15], low density lipoproteins (LDL) by following the procedure of [15], high density lipoprotein (HDL) by HDL Cholesterol Precipitant method [16] and triglycerides level by liquid triglycerides (GPO–PAP) method [15].

### Serum glucose and insulin levels

Glucose concentration of individual rat in each study was determined by GOD-PAP method as described by [17], whereas insulin level was estimated by the method of [18].

### Antioxidant status

Glutathione contents were determined following the protocols as described by [19]. The colored product of GSH + DTNB in the protein free supernatant was measured at 412 nm and expressed as nmol/mg protein. Similarly, indicator of lipid peroxidation i.e. thiobarburic acid reactive species (TBARS) was also estimated [20].

### Statistical analysis

The piled data were analyzed for the level of significance (*p* ≤ 0. 05) through CRD one factor factorial and significance among the treatments were determined through LSD Test by using Statistical Package (Costat-2003, Co-Hort, v 6.1.) Moreover, Correlation analysis, Principle component analysis and Dendrogram were also plotted by using XLS-Stat software to estimate the correlation among the different study parameters [21].

## Results and discussion

### Extraction yield

**Fig. 1:**
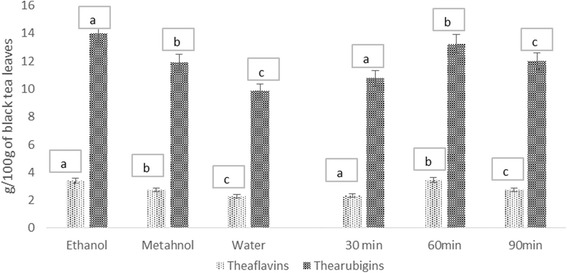
Absolute values for Extraction yield for isolated theaflavins and thearubigins from Black tea (QI-Men)

It is evident from fig that the extraction yield of both theaflavins and thearubigins were significantly affected by type of solvents (*p* ≥ 0.001) and time of extraction (*p* ≥ 0.002). The maximum theaflavins and thearubigins yield (3.50 ± 0.31 g/100 g & 13.18 ± 1.07 g/100 g) were observed in ethanolic extract whilst minimum in water extract (2.32 ± 0.13 & 9.75 ± 0.42 g/100 g), respectively. Extraction efficiency was also influenced by time and highest yield for theaflavins and thearubigins was obtained at 60 min 3.39 ± 0.17 g/100 g and 13.01 ± 0.57 g/100 g, respectively (Fig. 1).

### HPLC quantification of individual fractions of theaflavins

The HPLC analysis revealed that both solvent (*p* ≥ 0.001) and time of extraction (*p* ≥ 0.0031) imparted momentous effect on theaflavins fractions. Means in fig indicated that the ethanolic extract showed highest values for all the theaflavin fractions i.e. TF1, TF2A, TF2B & TF3 followed by methanolic and water extracts and among time intervals maximum values for all the fractions were observed at 60 min and minimum at 30 min (Table 2).

**Table 2:** HPLC characterization of isolated theaflavin

Solvents	Time			Means
	30 min	60 min	90 min	
Theaflavin1 (mg/g)				
Ethanol	2.01 ± 0.03	2.42 ± 0.01	2.11 ± 0.04	2.18 ± 0.06a
Methanol	1.89 ± 0.05	2.03 ± 0.03	1.96 ± 0.01	1.96 ± 0.03b
Water	0.562 ± 0.006	0.933 ± 0.003	0.761 ± 0.009	0.751 ± 0.001c
	1.48 ± 0.001c	1.79 ± 0.006a	1.61 ± 0.004b	
Theaflavin2A (mg/g)				
Ethanol	3.01 ± 0.36	3.92 ± 0.03	3.31 ± 0.01	3.41 ± 0.03a
Methanol	2.69 ± 0.02	3.12 ± 0.06	2.91 ± 0.03	2.90 ± 0.09b
Water	0.963 ± 0.003	1.45 ± 0.03	1.12 ± 0.02	1.17 ± 0.03c
	2.22 ± 0.02c	2.83 ± 0.05a	2.44 ± 0.03b	
Theaflavin 2B (mg/g)				
Ethanol	1.26 ± 0.02	1.92 ± 0.03	1.59 ± 0.02	1.59 ± 0.03a
Methanol	0.981 ± 0.06	1.22 ± 0.05	1.13 ± 0.07	1.11 ± 0.09b
Water	0.691 ± 0.04	1.03 ± 0.01	0.878 ± 0.03	0.864 ± 0.006c
	0.971 ± 0.001c	1.39 ± 0.03a	1.18 ± 0.06b	
Theaflavin 3 (mg/g)				
Ethanol	2.99 ± 0.03	3.61 ± 0.06	3.36 ± 0.02	3.32 ± 0.06a
Methanol	2.05 ± 0.06	2.96 ± 0.04	2.41 ± 0.09	2.47 ± 0.01b
Water	0.998 ± 0.004	1.12 ± 0.01	1.05 ± 0.04	1.04 ± 0.03c
	2.01 ± 0.09b	2.56 ± 0.03a	2.27 ± 0.09a	

Values are mean ± SEM (*n* = 03). Values in same column within each parameter with different letters were significantly different from each other

### In vitro pancreatic lipase (PL) inhibitory activity of black tea flavonoids

From Table 3 it was elucidated that the both theaflavin and thearubigins caused significant (*p* ≥ 0.001) dose dependent inhibition in pancreatic lipase activity. It is worth mentioning that among the solvents ethanolic extracts of both compounds showed more potent effect then that of ethanol and water. Moreover, highest inhibition was observed for theaflavin ethanolic extract followed by methanolic and water. In general, theaflavin showed more potent inhibition in comparison with thearubigins and in solvents the order is ethanolic extract ≥ methanolic extract ≥ water extract. Pancreatic lipase is major enzyme which plays key role in the absorption and further hydrolyzing the triacylglycerol to their respective components i.e. 2-monoacylglycerol and fatty acids which is mandatory for dietary fat absorption. It is evident from results that both theaflavins and thearubigins caused significant reduction in the activity of this enzyme which might be associated with their impact on hydrolysis of dietary fat, consequently decreasing the subsequent intestinal absorption of lipolysis products and explain possible mode of action for its lipid related abnormalities managing perspective.

**Table 3:** Pancreatic lipase inhibitory activity (%) of ethanolic, methanolic and water fractions of theaflavins and thearubigins

Concentration (μg/mL)	% pancreatic inhibitory activity						Means
	TFW	TFM	TFE	TRW	TRM	TRE	
0	8 ± 0.05	12 ± 0.02	14 ± 0.84	6 ± 0.1	9 ± 0.3	11 ± 0.73	10.00 ± 0.06 g
100	22 ± 1.6	26 ± 0.9	32 ± 1.2	15 ± 0.4	18 ± 0.6	24 ± 1.4	22.83 ± 0.89f
200	36 ± 1.8	44 ± 1.5	52 ± 2.1	30 ± 0.4	32 ± 2.1	41 ± 2.1	39.17 ± 1.26e
300	42 ± 1.2	52 ± 3.1	63 ± 2.5	35 ± 1.1	48 ± 2.6	52 ± 2.3	48.67 ± 2.26d
400	50 ± 3.3	62 ± 2.7	72 ± 4.5	40 ± 2.5	52 ± 4.1	59 ± 2.8	55.83 ± 2.44c
500	55 ± 2.9	78 ± 5.4	82 ± 3.7	49 ± 2.2	58 ± 3.7	65 ± 4.2	64.50 ± 4.53b
600	65 ± 3.1	82 ± 4.5	91 ± 5.1	62 ± 4.5	75 ± 3.4	81 ± 6.7	76.00 ± 5.40a
Means	39.71 ± 1.26e	50.86 ± 3.66b	58.00 ± 4.36a	33.86 ± 1.6f	41.71 ± 2.58d	47.57 ± 3.75c	

Values are mean ± SEM (*n* = 03). Values in same column within each parameter with different letters were significantly different from each other

*TFW* Theaflavin water extract, *TRW* thearubigins water extract, *TFM* Theaflavin methanolic extract, *TRM* thearubigins methanolic extract, *TFE* Theaflavin ethanolic extract, *TRE* Thearubigins ethanolic extract

### Feed and drink intakes

**Fig. 2:**
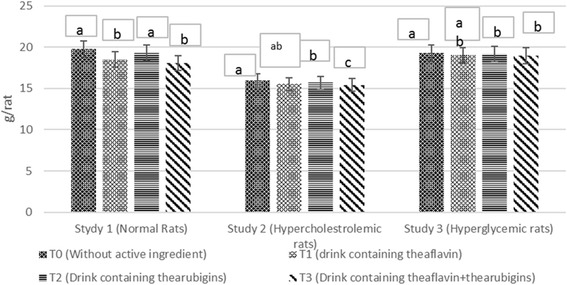
Agglomerative hierarchical clustering (AHC) of different studied attributes (dendogram)

**Fig. 3:**
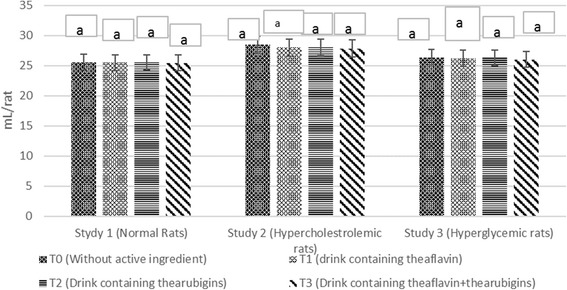
PCA analysis of the studied attributes showing the % share of two main factors (F1 and F2) in total variability

The means in Fig. 2 indicated higher feed intake in T_0_ (control drink) trailed by T_2_ (thearubigins supplemented) T_1_ (theaflavin supplemented) and T_3_ group (theaflavin+thearubigins supplemented) in all studies. It is evident that the experimental groups showed significant less feed consumption as compared to control. However, gradual increase was observed in feed intake with passage of time in all groups, more in T_0_ as compared to, T_1,_ T_2_ and T_3_ groups. Means regarding drink intake of rats (Fig. 3) in all studies disclosed progressive increase in drink intake during the entire trial with non-momentous differences among the treatments thus indicating the product acceptability. The same trend regarding feed & drink intakes were observed during subsequent trial. The results of instant study are supported by the findings of Yang et al. [22] noticed less feed consumption in rats administrated on black tea bioactive supplemented drinks. Likewise, Alshatwi et al. 16] observed less feed intake in hypercholesterolemic rats after theaflavin and thearubigins ingestion. They explicated that theaflavin and thearubigins improved the feed efficiency thus modulate the food consumption.

### Body weight

**Fig. 4:**
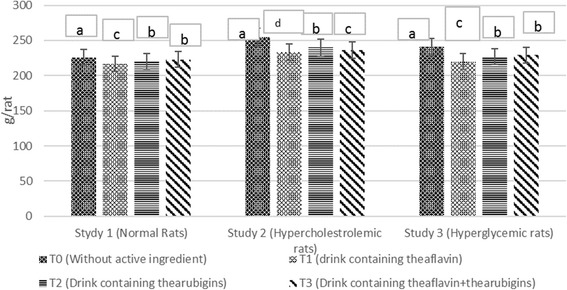
Diet plan used in all studies

Body weight of rats in all studies (Fig. 4) elucidated significant differences due to treatments. The weight gain increase significantly with passage of time however, maximum increase was observed in control drink (T_0_) feed group showed maximum weight gain followed by T_1_, T_2_ whilst minimum in T_3_ in all studies. Means for final body weight (Table 4) at the termination of study depicted substantial differences among the treatments. In study 1, the highest weight was observed in T0 group (225 ± 4.21 g/rat) trailed by T2 (220.48 ± 5.52 g/rat) and T3 (218.22 ± 7.25 g/rat) whilst minimum in T_1_ (215.30 ± 4.14 g/rat). Likewise, in study II, the T_0_ (255 ± 7.51 g/rat) group had more weight as compared to T_2_ (242.28 ± 6.12 g/rat), T_3_ (237.12 ± 6.91 g/rat) and T_1_ (231.74 ± 7.12 g/rat). Body weight at the termination of study III in T_0_ (243 ± 6.21 g/rat) was significantly higher than T_2_ (232.94 ± 5.34 g/rat) T_3_ (230.34 ± 7.21 g/rat) and T1 group (224.78 ± 7.23 g/rat), respectively during the entire trials. It is deduced from the results that theaflavin based experimental drink showed highest weight lost tendency in all study followed by T3 and T1 as compared to control. However, maximum decline in weight was observed in hypercholestrolemic rats (Study II) as 9.12, 4.99 and 7.01% by T1, T2 and T3, respectively as compared to control. Same tend was observed in trial II that validate the results.

**Table 4:** Effect of theaflavin and thearubigins and their combination based intervention on body weight serum triglycerides (TG), low density lipoprotein (LDL-C), high density lipoprotein (HDL-C), total cholesterol (TC), glucose and insulin in normal, hypercholestrolemic and hyperglycemic rats at the termination of the study

Parameters	Baseline values	Treatments	Normal rats (Study I)		Hypocholestrolemic rats (Study II)		Hyperglycemic rats (Study III)	
			Trial 1	Trial 2	Trial 1	Trial 2	Trial 1	Trial 2
Body weight (at termination of study)	128 ± 10.25	T_0_ (Control)	225 ± 4.21a	228 ± 5.01a	255 ± 7.51a	253 ± 6.25a	243 ± 6.21a	240 ± 7.25a
		T_1_	215.30 ± 4.14c	216.13 ± 5.21c	231.74 ± 7.12d	227.17 ± 6.12d	224.78 ± 7.23c	223.24 ± 9.21c
		T_2_	220.48 ± 5.52ab	220.21 ± 4.13ab	242.28 ± 6.12b	240.20 ± 5.43b	232.94 ± 5.34ab	228.74 ± 6.21ab
		T_3_	218.22 ± 7.25b	217.30 ± 8.42b	237.12 ± 6.91c	235.57 ± 5.62c	230.34 ± 7.21b	228.02 ± 5.21b
TC (mg/dL)	77.80 ± 3.56	T_0_ (Control)	80.25 ± 4.21	83.27 ± 5.01	148 ± 7.51a	145 ± 6.25a	97 ± 3.21a	95 ± 4.25a
		T_1_	77.24 ± 4.14	80.81 ± 5.21	131.67 ± 7.02d	129.58 ± 6.14d	89.60 ± 5.21c	87.35 ± 3.21c
		T_2_	78.68 ± 5.52	82.08 ± 4.13	141.50 ± 6.12b	136.53 ± 5.43b	92.96 ± 5.34b	90.65 ± 6.21b
		T_3_	77.87 ± 2.25	81.23 ± 3.41	137.65 ± 6.91c	134.60 ± 5.62c	91.05 ± 7.21b	90.00 ± 5.21b
LDL-C (mg/dL)	26.56 ± 3.56	T_0_ (Control)	30.08 ± 1.26	28.09 ± 2.31	61.74 ± 2.31a	59.45 ± 3.13a	48.36 ± 3.51a	46.86 ± 4.13a
		T_1_	28.91 ± 1.83	26.95 ± 1.98	52.94 ± 3.92d	50.47 ± 2.13d	43.31 ± 2.91c	41.19 ± 2.33c
		T_2_	29.69 ± 1.32	27.54 ± 1.96	57.02 ± 4.14b	54.61 ± 3.90b	45.93 ± 2.92b	44.00 ± 2.57b
		T_3_	29.11 ± 1.64	26.97 ± 1.43	55.42 ± 2.51c	52.21 ± 3.12c	44.50 ± 3.63b	43.05 ± 3.54b
HDL-C (mg/dL)	32.56 ± 3.56	T_0_ (Control)	35.76 ± 2.35	37.47 ± 3.35	57.67 ± 4.11b	59.22 ± 3.62b	40.55 ± 1.09b	41.56 ± 2.03b
		T_1_	36.83 ± 2.61	38.47 ± 1.09	60.70 ± 5.32a	62.45 ± 5.62a	42.31 ± 3.22a	43.33 ± 4.54a
		T_2_	36.22 ± 2.51	38.04 ± 2.11	58.96 ± 5.12b	60.58 ± 3.63b	41.28 ± 3.12ab	42.35 ± 2.90ab
		T_3_	36.76 ± 2.11	38.31 ± 1.61	60.06 ± 3.92a	61.80 ± 4.11a	42.17 ± 3.91a	43.12 ± 2.80a
TG (mg/dL)	58.26 ± 3.56	T_0_ (Control)	65.73 ± 5.21	67.47 ± 4.23	98.88 ± 7.02a	95.01 ± 6.52a	78.99 ± 5.13a	76.14 ± 6.31a
		T_1_	63.76 ± 2.03	65.44 ± 4.12	90.43 ± 7.05d	87.45 ± 6.35d	73.82 ± 5.10c	70.79 ± 3.19c
		T_2_	64.68 ± 3.20	66.11 ± 4.01	95.13 ± 5.81b	91.19 ± 6.02b	76.01 ± 6.21b	73.09 ± 5.69b
		T_3_	64.01 ± 3.08	65.88 ± 4.02	92.26 ± 7.01c	88.35 ± 5.03c	74.94 ± 4.15b	71.56 ± 5.21b
Glucose (mg/dL)	79.63 ± 3.72	T_0_ (Control)	85.13 ± 6.23	87.60 ± 5.03	100.34 ± 8.52a	98.02 ± 7.13a	135.19 ± 9.99a	140.91 ± 8.54a
		T_1_	82.57 ± 4.52	85.10 ± 6.12	93.95 ± 8.25b	90.76 ± 8.56b	122.85 ± 9.63c	128.49 ± 9.25c
		T_2_	82.61 ± 6.02	85.84 ± 5.86	92.13 ± 6.98b	94.41 ± 7.45b	126.04 ± 9.45b	132.18 ± 7.46b
		T_3_	81.74 ± 3.25	84.22 ± 4.86	91.49 ± 7.84c	88.44 ± 6.98c	120.28 ± 8.51d	123.67 ± 6.35d
Insulin μU/Ml	6.23 ± 0.56	T_0_ (Control)	7.59 ± 0.65	8.74 ± 0.70	9.56 ± 0.36b	10.89 ± 0.35b	12.02 ± 1.01bc	13.37 ± 1.12bc
		T_1_	7.81 ± 0.15	9.01 ± 0.32	10.03 ± 0.92a	11.33 ± 0.45a	12.97 ± 1.22a	14.39 ± 1.32a
		T_2_	7.75 ± 0.61	8.94 ± 0.81	9.83 ± 0.94a	11.19 ± 0.84a	12.47 ± 1.21b	13.85 ± 1.23b
		T_3_	7.84 ± 0.45	9.10 ± 0.41	10.09 ± 0.15a	11.59 ± 0.86a	13.02 ± 1.02a	14.55 ± 1.13a
Glutathione mg/L	49.01 ± 2.01	T_0_ (Control)	48.49 ± 3.12c	49.51 ± 2.98c	38.27 ± 3.12c	39.45 ± 3.15d	41.23 ± 2.09c	40.28 ± 3.06c
		T_1_	50.43 ± 2.92ab	51.97 ± 3.15ab	41.72 ± 2.35b	43.40 ± 3.55b	44.22 ± 3.47b	43.59 ± 3.12b
		T_2_	49.49 ± 2.10b	51.41 ± 2.12b	40.23 ± 2.98bc	41.81 ± 2.12c	43.30 ± 3.71bc	42.36 ± 3.51bc
		T_3_	51.47 ± 1.21a	52.46 ± 3.91a	45.73 ± 2.98a	46.93 ± 4.12a	48.24 ± 4.01a	46.99 ± 4.11a
TBARS μmol/L		T_0_ (Control)	6.97 ± 0.02a	7.51 ± 0.05a	10.15 ± 0.07a	9.30 ± 0.09a	8.08 ± 0.10a	8.63 ± 0.14a
		T_1_	6.57 ± 0.01b	7.09 ± 0.51b	8.31 ± 0.42c	7.71 ± 0.01c	6.93 ± 0.42c	7.44 ± 0.53c
		T_2_	6.76 ± 0.04a	7.28 ± 0.01a	9.64 ± 0.02b	8.88 ± 0.01b	7.75 ± 0.03b	8.21 ± 0.02b
		T_3_	6.63 ± 0.01b	7.14 ± 0.03b	9.26 ± 0.01bc	8.54 ± 0.05bc	7.51 ± 0.04b	7.85 ± 0.03c

Values are mean ± SEM (*n* = 05). Descriptive Statistics were applied to check the overall behavior of the study parameter to elaborate the effect of treatments on selected parameter of rats. Values in same column within each parameter with different letters were significantly different from each other

*NS* Non Significant

Study I: Normal rats

Study II: Hypercholestrolemic rats

Study III: Hyperglycemic rats

T_0_: Control drink (without active ingredients)

T_1_: Drink containing Theaflavin

T_2_: Drink containing Thearubigins

T_3_: Drink containing Theaflavin + Thearubigins

The obesity lowering effect of tea nutraceutical based interventions in current study is in harmony with the earlier findings of Huang et al. [23, 24] reported 48.8% reduction in weight of obese CF-1 mice after administrating 0.2% black tea extract. They suggested that black tea antioxidants like theaflavin and thearubigins have potential to prevent fat oxidation and decrease the absorption of nutrients in gastrointestinal track. Moreover, they manage the energy consumption thus prevent LDL deposition and obesity. Likewise, Uchiyama et al., [22] also reported 44.2% reduction in weight of rats after theaflavin and thearubigins administration. They deduced that theaflavin inhibits the pancreatic lipase activity alongside intestinal lipid absorption thus attenuates the gain in weight. Similar sort of findings regarding theaflavin ability for weight reduction was noticed by Lin and Lin-Shiau [25] they highlighted that the compounds containing galloyl moiety suppressed the post pyrandial hypertriacylglycerolemia by slowing down the triacylglycerol absorption through the inhibition of pancreatic lipase. Theaflavin contains two digallate groups thereby has more potential for weight management than thearubigins. On molecular level, different enzymes played a key role to regulate lipid metabolism nevertheless, fatty acid synthase (FAS) is a considerate factor. Its Imbalance triggers the cascade of certain maladies like obesity, cardiovascular complications and cancer insurgence. The FAS inhibitors may help in weight management and in this context, black tea theaflavin is a promising ingredient. It blocks FAS through the deactivation of PI-3 K/AKT/ Sp-1 pathway owing to galloyl moiety [26].

### Lipid profile

**Fig. 5:**
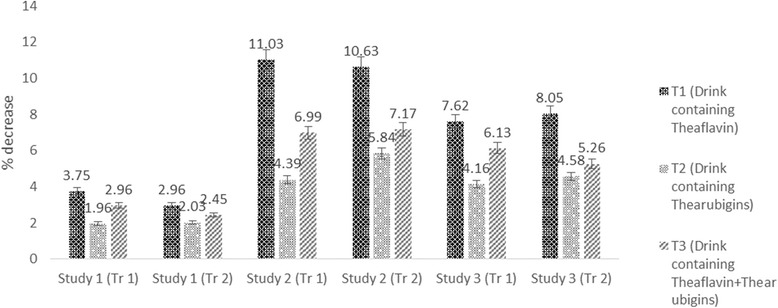
Drink plan used in all studies

**Fig. 6:**
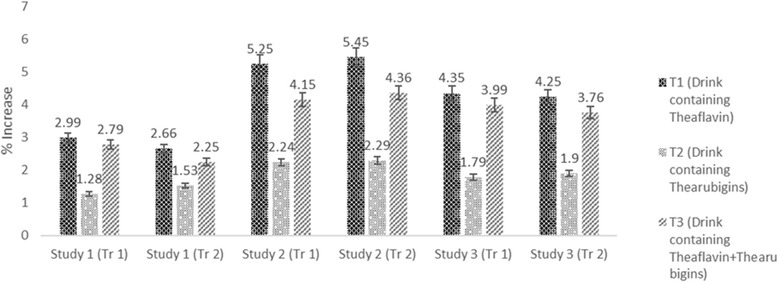
Average body weight at the termination of all studies

**Fig. 7:**
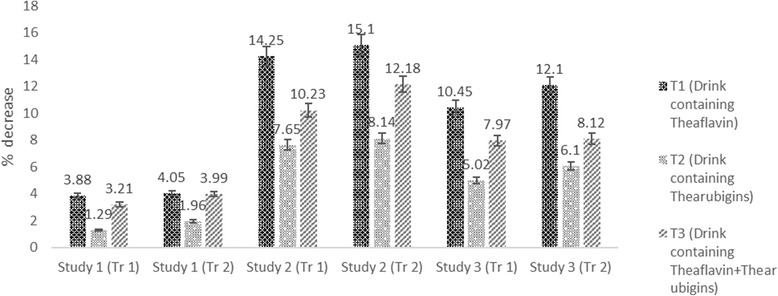
Percent reduction in cholesterol in all studies (both trials) by administration of theaflavin, thearubigins and combination of theaflavin & thearubigins based drink in rats

The Mean values in Table 4 indicated that treatments imparted significant variations on cholesterol in all studies. In study I, maximum cholesterol was observed in T_0_ (80.25 ± 4.21 mg/dL) followed by T_2_ (78.68 ± 5.52 mg/dL) and T_3_ (77.87 ± 2.25 mg/dL) groups however, minimum level (77.24 ± 4.14 mg/dL) in T_1_. Means for cholesterol in study II indicated maximum value for T_0_ (148 ± 7.51 mg/dL) that momentously reduced to 131.67 ± 7.02_,_ 141.50 ± 6.12 and 137.65 ± 6.91 mg/dL in T_1,_ T_2_ and T_3,_ respectively. Similarly, in study III, high cholesterol value 97 ± 3.21 mg/dL was noticed in T_0_ followed by T_2_ 92.96 ± 5.34 mg/dL and T_3_ 91.05 ± 7.21 mg/dL whilst lowest value 89.60 ± 5.21 mg/dL in T_1_. It is evident from the results that theaflavin based dietary intervention imparted maximum reduction as compared to thearubigins and theaflavin+thearubigins based intervention in all studies. However, effect was more pronounced in study II (hypercholestrolemic rats) with (11.03, 4.39 & 6.99% reduction in cholesterol with T_1,_ T_2_, & T_3,_ respectively (Fig. 5). Theaflavin and thearubigins based dietary interventions enhance HDL in all studies however, momentous effect was only observed in study II & III. In this context, T_1_ imparted maximum increase in HDL in study II & III as 5.25 & 4.35% followed by T_3_ (4.15 and 3.99%) and T_2_ (2.24 and 1.79%), respectively (Fig. 6). It is obvious from Table 3 that treatments imparted significant effect on LDL in all studies accepts study I. Among the all treatments, theaflavin based drink (T_1_) exhibited highest LDL lowering ability in all studies as compared to control and recorded LDL values were 28.91 ± 1.83, 52.94 ± 3.92, 43.31 ± 2.91 mg/dL, in study I, II & III, respectively with 3.85, 14.25 & 10.45% LDL decline, respectively followed by T_3_ as 3.21, 10.23 & 7.97% whilst, minimum LDL reduction was recorded for T_2_ 1.29, 7.65 & 5.02% in Study, I, II & III, respectively (Fig. 7).

**Fig. 8:**
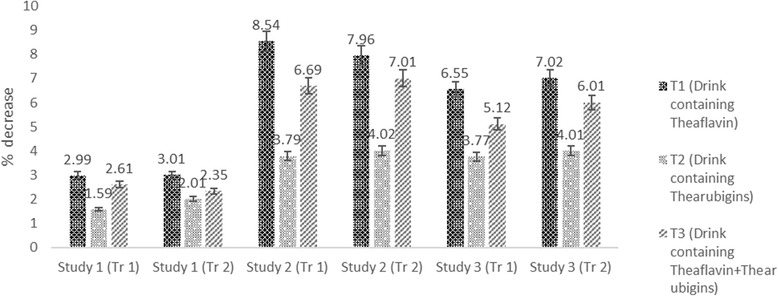
Percent increase in HDL in all studies (both trials) by administration of theaflavin, thearubigins and combination of theaflavin & thearubigins based drink in rats

Triglycerides levels affected significantly by treatment in all studies. Means regarding triglycerides depicted highest triglyceride reduction in Study II by T_1_ (8.54%) followed by T_3_ (6.69%) & T_2_ (3.79%), respectively as compared to control (T_0_). Likewise, in study III, T_1_ showed maximum reduction 6.55% trailed by T_3_ 5.12% and T_2_ 3.77%, respectively (Fig. 8). The results regarding lipid profile of trial II were synchronized with the above results thus enhance the authenticity of research.

### Indicators for lipid peroxidation (oxidative stress)

The means regarding indicators of oxidative stress revealed that the tested drinks significantly affected the glutathione and TBARS status of experimental animals in all studies. Owing to the onset of accelerated free radical generation, T_0_ group showed suppressed glutathione value as 45.12 ± 2.01 mg/L that significantly (*p* ≥ 0.001) uplifted to 48.19 ± 2.16, 46.91 ± 3.14 and 49.87 ± 5.12 mg/L in T_1,_ T_2_ and T_3_ groups. Likewise, momentous (*p* ≥ 0.000) response of experimental treatments was noticed for TBARS, the observed values in T_0_ was 7.33 ± 0.19 that differed significantly in T_1,_ T_2_ and T_3_ by 6.59 ± 0.12, 7.06 ± 0.02 and 6.80 ± 0.40 μmol/L (Table 4).

The findings of Uchiyama et al. [22] are in accordance with the current results; they noticed an inverse association between tea consumption and cholesterol level. They administrated high fat diet to rats along with theaflavin @ 500 and 1000 mg/kg body weight for 8 weeks. At the termination of study, total cholesterol of male wistar rats were reduced up to 21.10% as compared to control. They suggested theaflavin ability to inhibit pancreatic lipase activity is one of the mechanistic approaches behind this reduction. Previously, one of the researchers group Davies et al. [27] also observed reduction in the lipid profile of hypercholesterolemic adults after black tea consumption. They deduced that black tea polyphenols modulate the metabolic targets like satiety, thermogenesis and fat oxidation. Furthermore, adipocyte differentiation suppression and fatty acid uptake from the adipose tissues by tea antioxidants are also the cardinal factors in this context [28].

Likewise, Alshatwi et al. [16] noticed a momentous reduction in cholesterol, LDL and triglycerides in hypercholesterolemic rats treated with black tea polyphenols. They deduced that high fat diet caused increase in rats cholesterol level alongside hepatic abnormalities. The menace is tackled by black tea bioactive constituents that inhibit lipid peroxidation. Moreover, they influence the fecal excretion of fatty acids and sterols that helps to get rid of cholesterol. Black tea is a rich source of bioactive constituents i.e. theaflavin, thearubigins and catechins that act alone or in combinations against different ailments. However, theaflavin improves lipid abnormalities more efficiently by modulating the expression of different enzymes involved in lipid metabolism i.e. fatty acid synthesis (FAS) [29].

Dyslipidaemia is one of the process that can accelerates the atherosclerotic process resulting morbid consequences ultimately leading towards poor human health. The treatment choice for decreasing low-density lipoprotein cholesterol level and declining cardiovascular complications are exploiting statins however, patient’s intolerance, side effects as well as patient preference is focusing the drug industry to look for alternatives. In this context, functional foods and nutraceuticals ingredients are used because they are considered effective to reduce overall cardiovascular risk induced by dyslipidaemia. The mechanistic approach behind their ability to control dylslipediemia are yet to explore completely however, reducing 7a–hydroxylase, increasing cholesterol faecal excretion, decreasing 3-hydroxy-3-methylglutaryl-CoA reductase mRNA levels or decreasing secretion of very low-density lipoprotein(VLDL). These potentials benefits of health promoting ingredients suggest promotion of nutraceuticals based functional foods as a therapy to manage lipid related disorders [30].

Theaflavin imparts reduction in lipid related abnormalities by stimulating the cellular energy expenditure on mitochondrial level that hinders the weight gain. However, in nucleus, the expression of FAS may be suppressed by theaflavin that down regulate the EGF-receptor/ PI3K/Akt/Sp-1 signal transduction pathway thus inhibits the cellular lipogenesis and tissue growth. It also modulates the LDL receptors that facilitate in cholesterol and triglycerides reduction [31]. Another possible route by which theaflavin performed lipid lowering function may be the interference with cholesterol micellar solubilization. The cholesterol absorption is mediated in different steps including emulsification, hydrolysis of ester bond, micellar solubilization, reesterification in intestine and transport to lymphatic cell through chylomicron [32, 33]. Cholesterol micelle solubilization is inevitable for cholesterol transport due to its water insolubility. Theaflavin acts on the micells and induces changes in their structure that cause reduction in cholesterol resynthesis and alters its metabolisim. Theaflavin ability to change the micells structure is further illuminated by the research work of Ikeda et al. [29], they indicated that theaflavin decreases the in vitro micelle solubility of cholesterol in dose dependent manner. Likewise, Vermeer et al. [34] narrated that theaflavin particularly theaflavin-3-gallate hinders the incorporation of cholesterol in to mixed micelles after halting their formation thus regulates the intestinal cholesterol absorption.

LDL is major cholesterol carrying lipoprotein in plasma and mainly composed of apo-B100 protein (25%), cholesterol esters (74.96%) and triglycerides (< 1%). It also contains linoleate (polyunsaturated fatty acid) that combines to cholesterol esters make it susceptible to oxidation. The LDL oxidation considers as a key connection to the development of atherosclerosis. It initiates abnormal changes in the macrophage and combines with macrophage scavenger receptor, consequently foam cells are produced containing cholesterol that deposit inside the arteries. An array of evidences has proven the ability of black tea polyphenols especially theaflavin to reverse LDL oxidation by scavenging the free radicals, hindering the foam cell formation and deposition [35, 36].

### Glucose & Insulin Levels

**Fig. 9:**
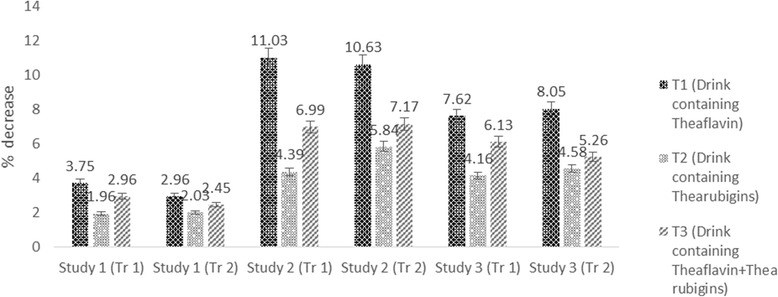
Percent decrease in LDL in all studies (both trials) caused by theaflavin, thearubigins and combination of theaflavin & thearubigins based drink in rats

The Mean values showed that glucose level in different groups of rats was significantly affected by treatments in all studies except for study I (Table 3). In study I, mean glucose values in T_0_, T_1,_ T_2_ and T_3_ groups were 85.13 ± 6.23, 82.57 ± 4.52, 82.61 ± 6.02 and 81.74 ± 3.25 mg/dL, respectively. In study II, T_0_ group illustrated highest glucose level 100.34 ± 8.52 mg/dL that substantially decreased to 91.49 ± 7.84 93.95 ± 8.25 and 94.41 ± 7.45 mg/dL in T_3_ (drink containing theaflavin+thearubigins)_,_ T_1_ (drink containing theaflavin) and T_2_ (drink containing thearubigins) groups, respectively_._ Likewise, high sucrose fed rats (study III) had maximum glucose level 135.19 ± 9.99 mg/dL in T_0_ group that reduced significantly by T_3_ (120.28 ± 8.51 mg/dL) followed by T_1_ (122.85 ± 9.63 mg/dL) and T_2_ (126.04 ± 9.45 mg/dL). It is also evident that theaflavin+thearubigins supplemented drink (T_3_) led to maximum glucose decline in all studies and highest was detected in study III as 11.03% (Fig. 9).

**Fig. 10:**
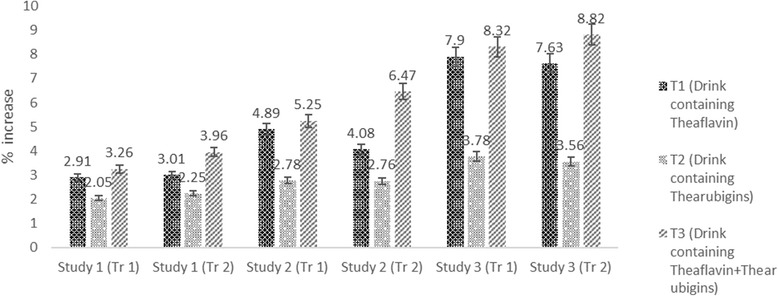
Percent diminish in triglycrides levels in all studies (both trials) caused by theaflavin, thearubigins and combination of theaflavin & thearubigins based drink in rats

The Mean values elucidated that treatments imparted non-significant effect on insulin in study I however, this trait was affected significantly in study II & III. In study II, the highest insulin level was recorded in T_3_ 10.09 ± 0.15 followed by T_1_ and T_2_ and T_0_ as 10.03 ± 0.92, 9.83 ± 0.94 & 9.56 ± 0.36 μU/Ml, respectively. Likewise trend was observed in in study III, highest elevation from 10.89 ± 0.35 to 11.59 ± 0.86 μU/Ml (8.32%) was noticed in T_3_ followed by 10.89 ± 0.35 to 11.33 ± 0.45 μU/Ml (7.90%) in T_1_ whilst minimum 10.89 ± 0.35 to 11.19 ± 0.84 μU/Ml (3.78%) in T_2_. It is revealed that functional drink containing theaflavin+thearubigins (T_3_) performed better against glucose related abnormalities than drinks containing theaflavin (T_1_) and thearubigins (T_2_) alone (Table 4 & Fig. 10).

The conclusions of Abeywickrama et al. [37] are in accordance with the instant exploration; they recorded 21.02% decline in blood glucose of diabetic rats consuming black tea @ 480 mg/kg. They concluded that black tea managed glucose related variables by multiple mechanisms including inhibition of α-glucosidase & α-amylase activity, reduction in intestinal glucose absorption, insulinomimetic action and antioxidant capacity. The effect of tea on carbohydrate hydrolyzing enzymes is also observed by Sharangi [38] reported significant decline in the activity of α-amylase by slowing the breakdown of starch thus attenuates sudden rise in glucose. Similar trend regarding glucose and insulin was observed in following trial. Similarly, Shoji and Nakashima et al. [39] tested the glucose lowering potential of black tea in type II diabetic mice KKAVTaJcI. The examined tea extract caused 36.01% reduction in blood glucose. They proposed that theaflavin and other polyphenols in black tea inhibited glucose uptake from intestinal epithelial Caco-2 cells, possessed α-glucosidase activity and reduced the hepatic glucose synthesis thus control the glucose malfunctioning.

One of the possible mechanisms showing black tea as hypoglycemic agent is its ability to modulate the activity of glucose transporters (GLUTs); major carriers that maintain the glucose homeostasis and require IRβ and AMPKR proteins for their translocations. Different bioevaluation trials indicated a linear association between high fat consumption and insulin resistance lead to hyperglycemia and hypoinsulinemia resulting type 2- diabetes [40–43]. The high fat diet increased the production of free fatty acids that enhanced plasma triglyceride level, suppressed insulin receptors thereby cause insulin resistance [43]. Moreover, the fructose or sucrose rich diets trigger abnormal glucose production that affect different hormones in plasma like adiponectin and intestine GLUT1 linked with insulin resistance. The high fat and sugar diets initiate the cascade of coronary complications, obesity and diabetes by disturbing glucose homeostasis and insulin sensitivity [42, 43]. There are sound evidences showing black tea ability to improve insulin resistance in both hypercholesterolemic and hyperglycemic models thus attenuates diabetic mellitus and obesity [43]. The stimulation of incretion hormones of the enteroinsular axis (EIA) in pancrease and enhanced activity of GIP & GLP-1 factors are the possible mechanisms by which black tea polyphenols improved the insulin secretion.

### Correlation analysis

**Fig. 11:**
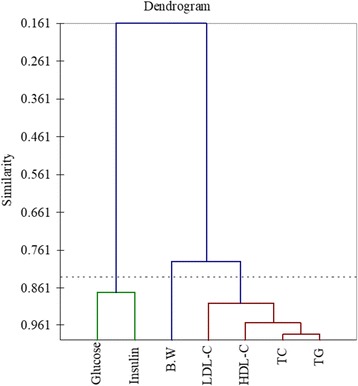
Percent reduction in glucose as compared to control in all studies

**Fig. 12:**
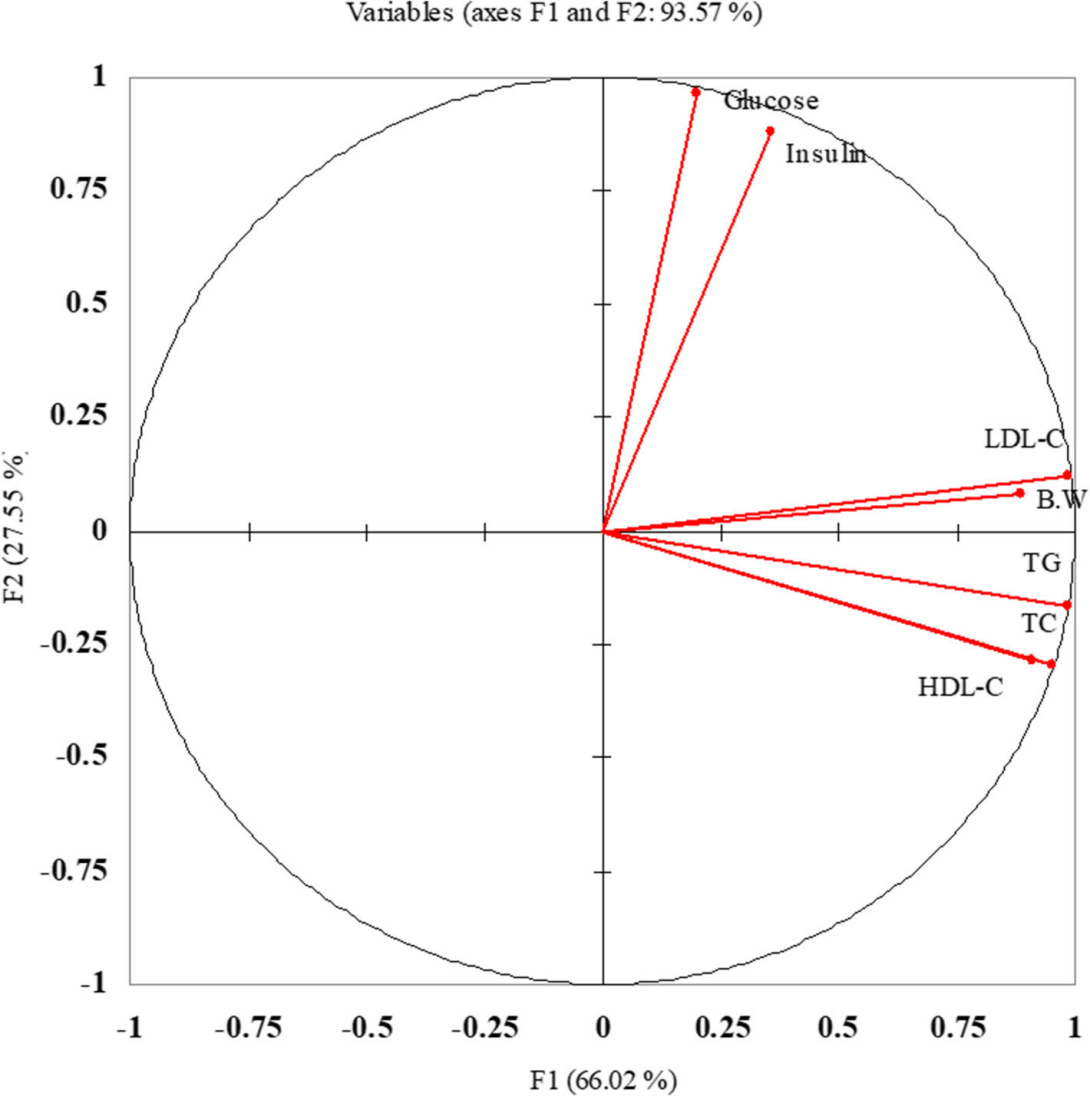
Percent increase in insulin as compared to control in all studies

Correlations analysis indicated significant presence of correlation among the different study parameters (Table 5 & Figs. 11 & 12). Significant positive correlation was observed for body weight with total cholesterol (*r* = 0.719***), LDL-C (*r* = 0.875***), HDL-C (*r* = 0.650***) & TG (*r* = 0.851***). Likewise, all the parameters relating to lipid profile were positively corelated with each other. However, a non-significant correlation was observed for glucose and insulin (*r* = 0.297*** & *r* = 0.275***) with lipid profile. Meanwhile glucose found positively corelated with insulin (*r* = 0.876***). Similarities among different studied attributes presented in Fig. 11 as dendrogram shows that studied attributes can be categorized in two major classes. One is indicators for lipid peroxidation i.e. Body weight, Total Cholesterol, LDL-C, HDL-C and TG attributes that have close similarity and the other are Insulin and Glucose that have significant positive relation. Likewise trend was observed from PCA graph.

**Table 5:** Pearson coefficient correlations (*r-values*) among different studied attributes

Variables	B.W	TC	LDL-C	HDL-C	TG	Glucose	Insulin
B.W	1.000						
TC	0.791***	1.000					
LDL-C	0.875***	0.898***	1.000				
HDL-C	0.650***	0.969***	0.856***	1.000			
TG	0.851***	0.985***	0.951***	0.939***	1.000		
Glucose	0.297 ns	−0.107 ns	0.322 ns	−0.127 ns	0.041 ns	1.000	
Insulin	0.275 ns	0.098 ns	0.450**	0.160 ns	0.201 ns	0.876***	1.000

*** and ** = significant at 0.001 and 0.01 levels respectively

*ns* non-significant

## Conclusions

It is deduced that the theaflavin and thearubigins functional drinks are valuable against lipid and glucose related abnormalities especially high cholesterol and LDL levels. Nonetheless, they are proven more effective in hyperchlestrolemic and hyperglycemic phase. It is noteworthy that drink containing theaflavin is more effective to attenuate lipid metabolism in comparison with thearubigins whereas their synergistic effect was more promising to tackle carbohydrate metabolism. Owing to the existence of theaflavin and thearubigins, respective functional drinks can be used for combating the lifestyle related maladies with special reference to hypercholesterolemia hyperglycemia. However, the mechanistic concerns which triggers these therapeutic responses should be demonstrated for better interpretation of their medicinal perspective. It is recommended that future in vivo studies should be conducted to evaluate the impact of black tea flavonoids on NADPH oxidase, HMG-CoA reductase activity and other related biomarkers to unveil the associated mechanism for their beneficial lipid peroxidation diminishing perspective.
